# Potential fire risks in South America under anthropogenic forcing hidden by the Atlantic Multidecadal Oscillation

**DOI:** 10.1038/s41467-022-30104-1

**Published:** 2022-05-04

**Authors:** Yanfeng Wang, Ping Huang

**Affiliations:** 1 Center for Monsoon System Research, Institute of Atmospheric Physics, Chinese Academy of Sciences, Beijing, China; 2 College of Earth Sciences, University of Chinese Academy of Sciences, Beijing, China; 3 State key Laboratory of Numerical Modeling for Atmospheric Sciences and Geophysical Fluid Dynamics, Institute of Atmospheric Physics, Chinese Academy of Sciences, Beijing, China

**Keywords:** Climate-change impacts, Atmospheric dynamics, Environmental impact

## Abstract

Fires in South America have profound effects on climate change and air quality. Although anthropogenic forcing has exacerbated drought and fire risks, the fire emissions and aerosol pollution in the southern Amazon and the Pantanal region showed a consistent long-term decrease during the dry season (August–October) between 2003 and 2019. Here, we find that the decreasing trend in fire emissions, mainly located in the non-deforested region, was associated with climatic conditions unfavorable for intensifying and spreading fires, including increased humidity and slower surface wind speed. These climatic trends can be attributed to weakening of the positive phase of the Atlantic Multidecadal Oscillation, which has strengthened the northeast trade winds within the region (3°S–13°N) and the northwest winds east of the Andes that transport more moisture into the southern Amazon and the Pantanal region. Our findings show the mitigating effects of weakening of the positive Atlantic Multidecadal Oscillation phase on human-induced intensification of fire risks in South America and warn of potentially increased risks of fires and aerosol pollution under intensified anthropogenic forcing in the future.

## Abstract (web summary)

Fire emissions in South America consistently decreased in 2003–2019, although anthropogenic forcing could exacerbate drought and fire risks. Here the authors find that the decreasing fires were associated with climatic conditions unfavorable for intensifying and spreading fires, led by the phase transition of the Atlantic Multidecadal Oscillation.

## Introduction

The world’s largest tropical rainforest and wetlands are located in South America: the Amazon rainforest and the Pantanal wetlands^1–3^. The Amazon rainforest stores about 100 Pg of carbon and is unsurprisingly recognized as a potential tipping point in the global climate system^1,4–6^. Unfortunately, the observed Brazilian Amazon rainforest cover had declined by almost 20% in 2019 compared with the forest cover in 1988^1,7–10^, while the Amazon dieback threshold to maintain the current climate equilibrium is estimated to be only 40%^1,9^.

Fire risks in South America are attracting considerable attention owing to their position at the intersection of the carbon cycle, climate, and ecosystems^1,9,11–14^. Fire has an important impact on ecosystems in South America by influencing the balance between savanna and rainforest^15^. It can also cause deforestation, destruction of biodiversity, economic losses, and human casualties^1,16–28^. The vast quantities of emissions associated with fires directly influence the concentrations of greenhouse gases (GHGs) in the atmosphere, and the burnt aerosols from fires, the main component of aerosols in the Amazon’s dry season, have a substantial influence on the regional air quality^21–25,27,29,30^. Positive feedback among drought, fire, and deforestation can further amplify the destructiveness of fires^8,11,31^.

The main threat of fire in this region lies in the southern Amazon and the Pantanal (SAP) region (3°–28°S, 40°–80°W). Anthropogenic forcing (e.g., aerosols, warming caused by GHG emissions, and changes in land use) would increase the fire risks in the SAP region^2,3,32,33^. However, both fires and aerosol optical depth (AOD) in the southern Amazon show a decreasing trend in the time period of around 2000–2010 ^20,34–36^. Previous studies suggested that a reduced deforestation rate could be the main contributor to the reduced incidence of fire in Amazonia for the time periods 2000–2007^35^ and 2001–2012 without 2005, 2007, and 2010^34^. However, the emissions in the deforested and non-deforested regions were not well separated, and thus the roles of other factors are not clear yet.

Moreover, the climate conditions dominate the variation in fire emissions in the non-deforested region by influencing its intensification and spread^11,20,37^. The fire-related climate conditions in South America can be influenced by the variations in the sea surface temperature (SST) in the Pacific and Atlantic oceans, such as the El Niño–Southern Oscillation and the Pacific Decadal Oscillation (PDO) in the Pacific and the Atlantic Multidecadal Oscillation (AMO) in the North Atlantic^20,38–48^. The North Tropical Atlantic SST is also used for the seasonal forecasting of the dry season fire risks in the western Amazon^48–50^.

In this study, we separate the amount of burnt carbon in the deforested and non-deforested regions in the dry season of 2003–2019 and find that the decreasing trend in fire emissions in the SAP region is mainly located in the non-deforested region. The decreasing fire emissions are related to the unfavorable climate conditions for fire emissions including the increased humidity and slower surface wind speed. Analyzing the Detection and Attribution Model Intercomparison Project^51^ (DAMIP) and reanalysis datasets, we reveal that the unfavorable climate conditions for fire emissions can be attributed to the weakening positive phase of the AMO during this period, and the anthropogenic forcing very likely enhances the climatic conditions favoring fire emissions in the SAP region.

## Results

### Changes in fire emissions in South America

**Fig. 1:**
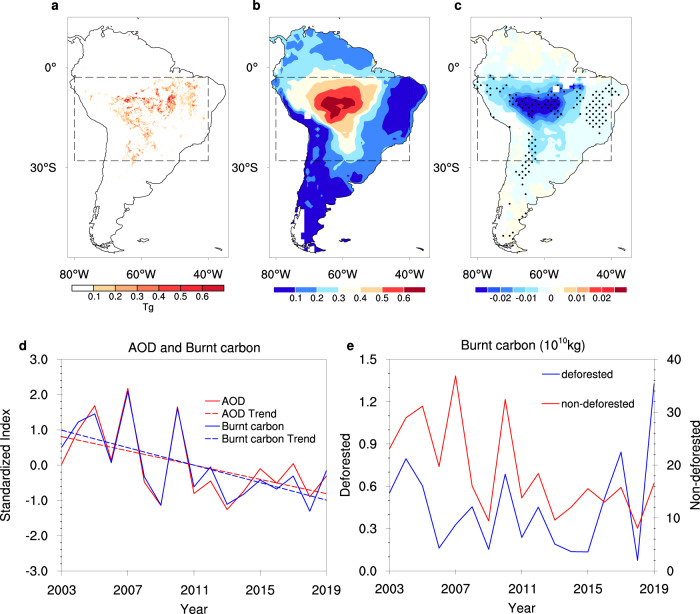
Burnt carbon and AOD in South America during the dry season in the time period 2003–2019. **a** Cumulative amount of burnt carbon and **b** the seasonal-mean AOD. **c** Long-term trend of the AOD (units: yr^−1^), where the stippling indicates passing the Mann–Kendall test at the 0.1 significance level. The boxes outlined by black dashed lines show the location of the SAP region. **d** Time series and trend of standardized regionally averaged AOD and cumulative burnt carbon from fires in the SAP region. **e** Burnt carbon in the deforested and non-deforested areas of the SAP region.

The critical fire-prone region of South America lies in the SAP region (3–28°S, 40–80°W; marked by the black box in Fig. 1a)^21–25,27^. The cumulative amount of burnt carbon in the SAP region during the period of 2003–2019 accounted for about 10% of the annual global fire emissions (Supplementary Table 1) and, in the dry season (August–October), 83–97% of the total burnt carbon emissions in South America (Supplementary Table 2). Based on the Global Fire Assimilation System (GFAS) dataset^52^, most (0.1° × 0.1°) grid cells where cumulative burnt carbon in the dry season during 2003–2019 is greater than 0.1 Tg are located in the SAP region (Fig. 1a). The satellite-derived seasonal-mean (August–October) AOD^53^ is greater than 0.5 in the western part of the SAP region (3–28°S, 40–80°W), with a similar pattern to that of burnt carbon (Fig. 1a, b).

Consistent with the previous studies^20,34,35^, the AOD within 0–33°S showed a pronounced decreasing tendency in the dry season in the period 2003–2019, with the largest rate exceeding 0.025 yr^−1^ (Fig. 1c). The long-term decreasing trends of the regionally averaged AOD (*P* = 0.04) and cumulative burnt carbon (*P* = 0.01) in the SAP region are consistent and their interannual variations are highly correlated (*r* = 0.97; *P* < 0.0001; Fig. 1d). To investigate the role of deforestation, we calculated the amount of burnt carbon in the deforested and non-deforested regions separately (see Methods)^54^ in the dry season of 2003–2019 based on the High-Resolution (30 m) Global Maps of 21st-Century Forest Cover Change (Fig. 1e)^55^. The amount of burnt carbon in the non-deforested region was more than ten times that in the deforested region. The amount of burnt carbon in the non-deforested region significantly decreased during the dry season in the time period 2003–2019 (trend = −10.65 Tg yr^−1^, *P* = 0.008), around decreasing 4% per year relative to the level at the beginning of this period. Specifically, the amount of burnt carbon related to deforestation peaked in 2019, when the total amount of burnt carbon was still relatively small (Fig. 1d, e). These results suggest that deforestation is not the main factor in the decrease in fire emissions during the time period 2003–2019.

### Changes in the related climate conditions

**Fig. 2:**
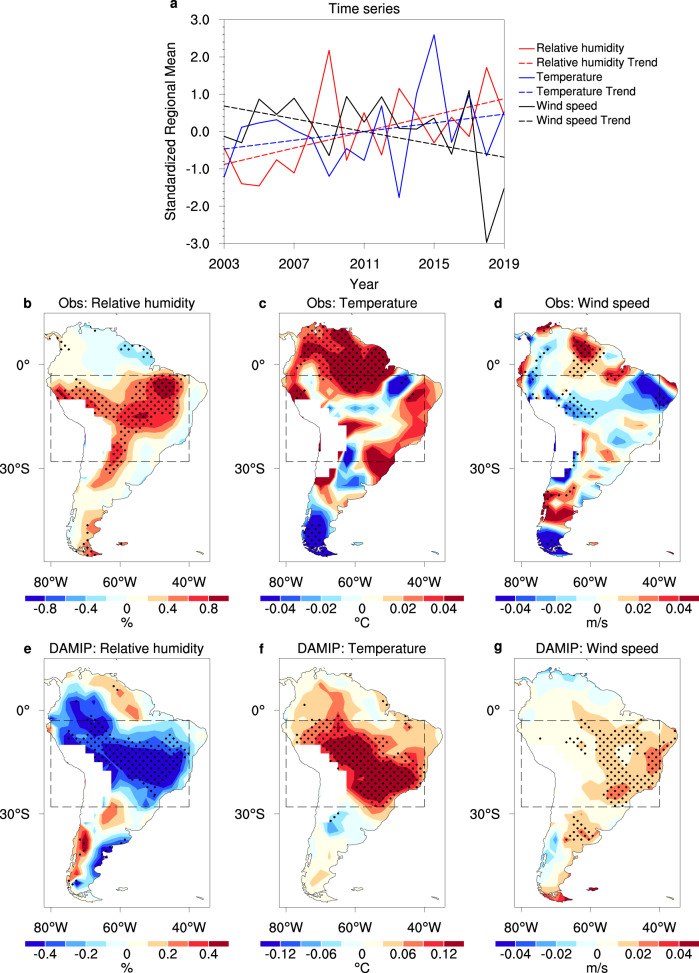
Changes in the climatic conditions related to fires in South America during the dry season. **a** Time series and trend of the observed standardized 925 hPa relative humidity, surface temperature, and surface wind speed in the SAP region during the time period 2003–2019. Long-term trends in the 925 hPa relative humidity (units: % yr^−1^), surface temperature (units: °C yr^−1^), and surface wind speed (units: m/s yr^−1^) from **b**–**d** the observations (2003–2019) and **e**–**g** anthropogenic forcing effects in the DAMIP dataset (2003–2014). Stippling indicates passing the Mann–Kendall test at the 0.1 significance level.

The variability of fire emissions in the non-deforested region is dominated by the efficiency of intensification and spread of fire, which is closely related to regional climate conditions^11,20,37^. The cumulative amount of burnt carbon in the SAP region during the dry season of 2003–2019 has a significant negative relationship with the low-level (925 hPa) relative humidity (*r* = −0.85, *P* < 0.001) and a positive relationship with the surface wind speed (*r* = 0.51, *P* = 0.04) (Fig. 2a). There was an apparent long-term increase in the low-level (925 hPa) relative humidity (*P* = 0.03) and a decrease in the surface wind speed (*P* = 0.09) in the SAP region, which would suppress the spread of fire^11,14,20,46,56^. Slight and insignificant warming of the surface temperature (*P* = 0.27) in the SAP region is also seen in Fig, 2a. However, the surface warming is expected to increase the drought risk^11,14,20,46^, and thus it could not contribute positively to the decrease in the fire emission. As a result, it is reasonable that the increased humidity and slower surface wind speed contributed to the coherent long-term decrease in the dry season fire emissions and aerosol pollution in the SAP region during the time period 2003–2019.

The regionally averaged relative humidity was significantly negatively correlated with the surface wind speed in the SAP region during 2003–2019 (*r* = −0.64, *P* = 0.006; Fig. 2a), implying that a large-scale variation in the circulation could enhance the transport of moisture into the SAP region and simultaneously slow the surface winds. Figure 2b–d shows the spatial pattern of the long-term trend of the 925 hPa relative humidity, surface temperature, and surface wind speed during the dry season of 2003–2019 in the SAP region. The increase in the relative humidity in the SAP region mostly exceeded 0.6% yr^−1^ (*P* < 0.1; Fig. 2b), with a few areas between 0.4 and 0.6% yr^−1^. The largest increase in the relative humidity was in the northeast of the SAP region, with a magnitude >0.8% yr^−1^, where the surface wind speed slowed at a rate >0.04 m s^−1^ yr^−1^ (*P* < 0.1, Fig. 2d). There were also opposite long-term trends for these two variables in northern Amazonia (3°S–13°N). The surface temperature in Fig. 2c shows a slight decrease in the center and northeast of the SAP region and a significant increase in the northwest of the SAP region and northern Amazonia.

The close relationship of the AOD with the relative humidity and the surface wind speed is further confirmed by their spatial correlation coefficients (Supplementary Fig. 4a, b). The AOD is negatively correlated with the local relative humidity in the SAP region and positively correlated with the surface wind speed (*P* < 0.05). The relationship of the AOD with the relative humidity and surface wind speed is reversed between northern Amazonia and the SAP region, similar to the long-term trends. This shows that the climate conditions, including the increase in the local humidity and the reduction in the surface wind speed, are very likely to suppress the risks of drought and fire in the SAP region.

### Role of anthropogenic forcing in the DAMIP

To clarify the roles of anthropogenic forcing or internal variability on these climate trends, we estimated the effects of anthropogenic forcing based on the historical simulations in the DAMIP^51^, which participates in Phase 6 of the Coupled Model Intercomparison Project (CMIP6)^57^. We defined the anthropogenic forcing effects as the differences between the simulations driven by natural plus anthropogenic forcing and by natural-only forcing. As simulated in the multi-model ensemble mean (Fig. 2e–g), the contribution of anthropogenic forcing was a reduction in the 925 hPa relative humidity and an increase in surface temperature and wind speed in the SAP region during the dry season in the time period 2003–2014. The decrease in the relative humidity derived from anthropogenic forcing was mostly greater than 0.1% yr^−1^ east of the Andes and 0.4% yr^−1^ in the southeast and northwest of the SAP region, where surface temperature warming was mostly >0.12 °C yr^−1^ (*P* < 0.1). The results in the single models are in good agreement with the multi-model ensemble mean (Supplementary Figs. 1–3).

The dry and hot environment, together with the intensified surface wind speed (*P* < 0.1) driven by anthropogenic forcing would markedly promote the spread of fire^3,14,32,33,56^. In turn, the fire would accelerate the transition of land cover from rainforest to savanna, further instigating the spread of fire^13,15,18^. The positive feedback on drought, fire, and deforestation might reinforce fire emissions. The DAMIP simulations suggest that the effects of anthropogenic forcing on the fire-related climate conditions (including the relative humidity and surface wind speed) are the opposite of the observed variations. Although anthropogenic forcing can cause significant surface warming in the SAP, the observed surface temperature in the SAP region showed an insignificant warming tendency in the dry season in the period 2003–2019 (Fig. 2a). The observed warming center lies in northern Amazonia, whereas the most significant warming caused by anthropogenic forcing is located in the SAP region. This indicates that internal climate variability processes could offset expected warming in the SAP region.

### Role of internal variability

For internal variability, both the PDO and AMO can influence the South American climate on an interdecadal timescale. However, the PDO mainly influences northern South America from December–May^40,42^, and the PDO from 2003 to 2012 featured an intensified negative phase, which should have increased the drought risk in the dry season in the SAP region^40,42^ in contrast to what was observed. The North Atlantic SST anomalies associated with the AMO could therefore be the driver of the fire-related climate conditions in the SAP region during the time period 2003 to 2019.

**Fig. 3:**
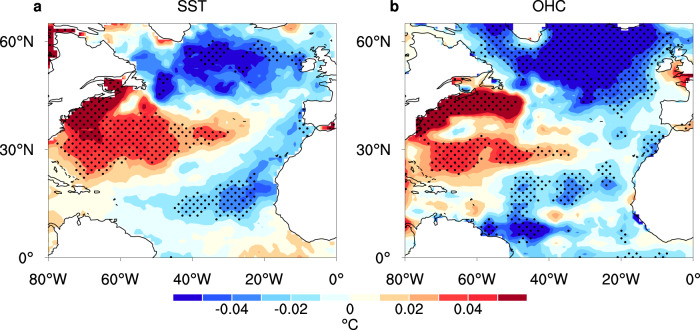
Long-term trends in the North Atlantic Ocean. Long-term trends in the **a** SST (units: °C yr^−1^) and **b** OHC (units: °C yr^−1^) in the North Atlantic Ocean during the dry season for the time period 2003–2019. Stippling indicates passing the Mann–Kendall test at the 0.1 significance level.

Figure 3 shows the long-term SST and the upper ocean heat content (OHC) tendencies in the North Atlantic Ocean during the dry season in the time period 2003 to 2019. The SST and OHC tendencies shared a similar spatial distribution, significantly decreasing in the North Tropical Atlantic as well as in the subpolar gyre and showing warming near North America^58–61^. These patterns were highly consistent with a trend of the AMO weakening from the peak of the positive phases^61–63^. The weakening of the positive AMO phase has been linked with the weakening of the Atlantic Meridional Overturning Circulation since 2004, as revealed in direct observations, proxies and model simulations^58–61^. Although sulfate aerosols could also influence the SST and OHC in the North Atlantic, the decrease in sulfate aerosols in the North Atlantic Ocean during the time period 2005–2015^64^ should have enhanced the positive phase of the AMO^65,66^, which is opposite of the observed trends.

**Fig. 4:**
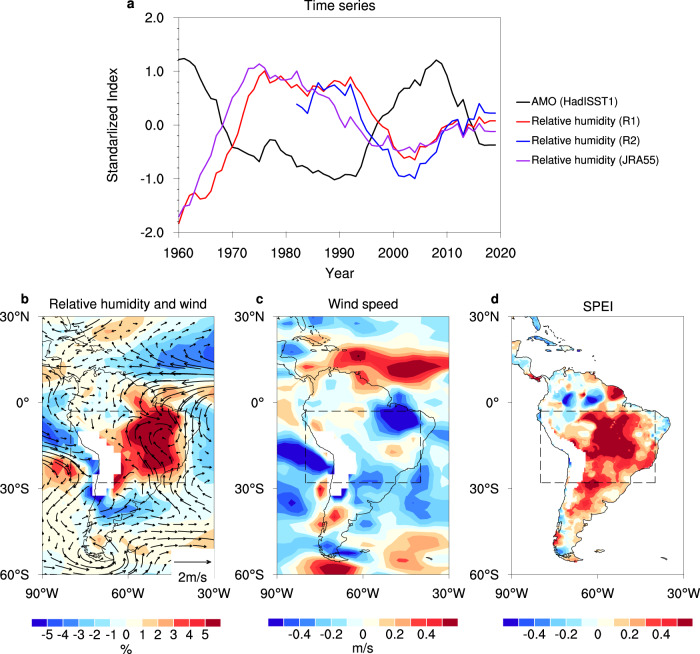
Linkages among the AMO and the 925 hPa relative humidity in the SAP region. **a** Standardized annual mean AMO index and the 925 hPa relative humidity (from the NCEP R1, R2, and JRA55 datasets) in the SAP region during the time period 1960–2019 after applying the 8-year running mean. The AMO index is detrended via the Residual method. **b**–**d** Differences in the 925 hPa relative humidity, 850 hPa wind vectors, surface wind speed, and the 5-month SPEI between the negative (1969–1996 and 2015–2019) and positive (1960–1968 and 1997–2014) phases of the AMO. The linear trends for all variables in the time period 1960–2019 have been removed.

The impacts of the AMO on the climate conditions in the SAP region are shown for a longer period from 1960 to 2019. Figure 4a shows the standardized annual mean AMO index and the 925 hPa relative humidity in the SAP (August to October) region after applying the eight-year running mean. The AMO index is significantly negatively correlated with the relative humidity (R1) in the SAP region (*r* = −0.82, *P* < 0.001) in the time period 1960–2019. Increasing relative humidity in the SAP region occurred during the weakening of the positive AMO phase (e.g., the time period 2003–2019) and vice versa. The relationship between the AMO and the SAP fire emissions was further verified in the significant correlation of the regionally averaged AOD in the SAP region with the North Atlantic SST and OHC during the dry season in the time period 2003–2019 (Supplementary Fig. 4).

Based on long-term observational records, we investigated the physical mechanism for the linkage between the weakening of the positive AMO phase and the suppressed fire risk in the SAP region. Figure 4b–d shows the analyzed differences in the relative humidity at 925 hPa, the wind vectors at 850 hPa, the surface wind speed, and the 5-month standardized precipitation evapotranspiration index (SPEI)^67^ between the negative (1969–1996 and 2015–2019) and positive (1960–1968 and 1997–2014) phases of the AMO.

There was an increased relative humidity, an intensified cyclonic circulation, a reduced surface wind speed, and an increased SPEI between the negative and positive phases of the AMO (Fig. 4b–d) in the SAP region, which corresponded to a mitigation of the local drought risk. The anomalies in the relative humidity were associated with the southward shift in the Atlantic Intertropical Convergence Zone and intensified northeast trade winds within the region (3°S–13°N) induced by the North Atlantic SST anomalies (Fig. 4b). Increased cross-equatorial flow from the North Tropical Atlantic to the northwest of South America intensified the northerly winds to the east of the Andes and brought more moisture into the SAP region, manifested as an increase in the relative humidity^41,68^ (Fig. 4b). Differences in the 925 hPa relative humidity and 850 hPa wind vectors between the negative and positive phases of the AMO were also verified using the 20th Century Reanalysis Project (1960–2015)^69^ and the JRA55 (1960–2019) datasets^70^ (Supplementary Fig. 5). The different climate anomalies between the negative and positive phases of the AMO over the longer-term permit us to infer a similar effect of the reduction in the positive phase of the AMO.

In addition to our analysis of the observational data, we also analyzed the historical simulations driven by observed climate forcing from 18 CMIP6 models^57^ during the dry season in the time period 1850–2014. The model results further show that the relative humidity increased in the SAP region during the negative phase of the AMO compared with the positive phase for all single models and the multi-model ensemble mean (Supplementary Fig. 6). In this time period, a relatively moist atmosphere combined with slower surface wind speed simultaneously suppressed the fire risks in the SAP region.

Some previous studies have suggested that intensified anthropogenic forcing, including future increases in GHG emissions and the clean-up of aerosols, could increase the risk of drought and fire in the SAP region in the dry season^3,66,71–73^. To examine this suggestion, we analyzed the changes in the fire-related climate conditions in the SAP region projected by the SSP585 simulations in eight CMIP6 models. Under intensified anthropogenic forcing, there were robust changes in the climate conditions in the dry season of the SAP region that were favorable for fire emissions (Supplementary Figs. 7–9), including reduced surface relative humidity and increased surface temperatures and surface wind speed. We conclude that higher risks of drought and fire will probably occur in the SAP region during the dry season under intensified anthropogenic forcing in the future.

## Discussion

In this study, we have shown the contribution of the weakening positive phase of the AMO to the coincident long-term decreasing trend of fire emissions and the AOD in the SAP region in 2003–2019. The variation of AMO can lead to climatic conditions unfavorable for intensifying and spreading fires, including an increase in humidity and slower surface wind speed during the dry season. During this period, the risk of drought and fire in the SAP region was temporarily mitigated by a weakening of the positive phase of the AMO. As the AMO is an internal fluctuation, its recent trend will not persist indefinitely. Therefore, the fire risk in the SAP region will be amplified by intensified anthropogenic forcing in the future. Human activities (e.g., deforestation) are also important factors in the risk of fire in the SAP region and deforestation has had an increasingly important role in fire emissions in recent years^2,3,11^. With a potential increased risk of drought in the future^3,20,72^, it is necessary to avoid a further increase in the risk of fire caused by human activities. The threshold of Amazon collapse is considered to be a loss of about 40% of its forested area to avoid reaching the potential tipping point^1,9^. Careful governance for fire prevention and rainforest protection is essential in the future.

## Methods

### Observational data

We used daily fire-induced burnt carbon data from the Global Fire Assimilation System (GFAS)^52^ and a combination of two monthly satellite-derived AOD datasets (MYD08_M3 and MOD08_M3) from the Moderate Resolution Imaging Spectroradiometer (MODIS) onboard the Terra/Aqua satellites^53^ to estimate the variability of fire emissions and aerosol pollution in South America. The cumulative monthly amount of burnt carbon was obtained from the sum of the daily burnt carbon emissions. The deforestation data were from the High-Resolution (30 m) Global Maps of 21st-Century Forest Cover Change^55^.

We obtained the observed surface temperature, surface wind speed, and 850 hPa wind vectors for the time period 1960–2019 from the NCEP Reanalysis 1 (R1) dataset^74^. We used three observational datasets for the 925 hPa relative humidity: the NCEP R1 dataset^74^ and the Japanese 55-year Reanalysis (JRA55) dataset^70^ for 1960–2019 and the NCEP Reanalysis 2 (R2) dataset^75^ for 1979–2019. We used the 925 hPa relative humidity from the R2 dataset for the time period 2003–2019 because the R2 dataset provides improved humidity data on the basis of R1^75^. To verify the reliability of the observational data, we also analyzed the 925 hPa relative humidity and 850 hPa wind vectors from the 20th Century Reanalysis V3 dataset^69^ and JRA55, respectively, for the time periods 1960–2015 and 1960–2019.

SST and subsurface sea temperature were respectively from HadISST1^76^ and EN4^77^. Along with HadISST1, the Kaplan Extended SST version2^78^ and the AMO time series from NOAA^79^ were also used to calculate the AMO indices in 1960–2019. The subsurface sea temperature averaged over the depth of 0–700 m defines the upper OHC.

### Model simulations

We used the historical simulations from the Detection and Attribution Model Intercomparison Project (DAMIP)^51^ of Phase 6 of the Coupled Model Intercomparison Project (CMIP6)^57^ to estimate the anthropogenic forcing effects, including the historical simulations driven by observed (historical) and natural-only (hist-nat) forcing. The eight models participating in DAMIP are BCC-CSM2-MR, CESM2, CanESM5, FGOALS-g3, GISS-E2-1-G, IPSL-CM6A-LR, MIROC6, and MRI-ESM2-0. We used three variables from the single simulation (r1i1p1f1) of DAMIP: the 925 hPa relative humidity, the surface temperature, and the surface wind speed. BCC-CSM2-MR does not provide surface wind speed data in DAMIP.

We used the historical simulations of the surface relative humidity for 18 CMIP6 models during the time period 1850–2014 to validate the mechanism of the impact of the AMO on South America. These 18 models were: ACCESS-ESM1-5, BCC-CSM2-MR, CESM2, CanESM5-CanOE, CanESM5, EC-Earth3-Veg, EC-Earth3, FGOALS-f3-L, GFDL-CM4, GFDL-ESM4, HadGEM3-GC31-LL, HadGEM3-GC31-MM, IPSL-CM6A-LR, MIROC6, MRI-ESM2-0, NorESM2-MM, SAM0-UNICON, and UKESM1-0-LL.

We also used the surface relative humidity, temperature, and wind speed from the historical and SSP585 simulations to estimate the influence of larger anthropogenic forcing in the future. Data from eight CMIP6 models were used: CESM2, CanESM5, FGOALS-g3, GFDL-ESM4, GISS-E2-1-G, IPSL-CM6A-LR, MIROC6, and MRI-ESM2-0.

### Techniques

#### Detrending and filtering

The AMO index is the regionally averaged detrended SST anomaly (SSTA) in the region (0–65°N and 0–80°W). To investigate the influence of detrending methods on the AMO series, we respectively removed the linear trend of the original (unsmoothed and not detrended) AMO index (https://psl.noaa.gov/data/timeseries/AMO/) in the time period 1960–2019 and 1856–2019 (see a brief discussion on the method in Supplementary Discussion and Supplementary Fig. 10). We also removed the linear trend from the HadISST1 and Kaplan SST datasets^78^ in the time period 1960–2019 to estimate the influence of the datasets on the AMO index. We then used a further two improved detrending methods based on the HadISST1 dataset: removal of (1) the global mean SSTA^80^ (referred to as GM); and (2) the regression of the SSTA on the yearly global mean SSTA^81^ (referred to as Residual) for the time period 1960**–**2019.

Referring to the method in Jones and Carvalho^41^, all the other variables were detrended using a linear trend over the period of 1960–2019 to remove possible anthropogenic forcing effects. We used the eight-year running mean as a low-pass filtering method to focus on the decadal/multidecadal variability.

#### Tests for trends and correlation coefficients

The correlation relationships were estimated using Pearson’s correlation coefficients and Student’s *t*-test. We obtained the long-term trend of the one-dimensional variable time series via least-squares linear regression and tested the significance of the trend via Student’s *t*-test (Figs. 1d, e and  2a). All the climate variables in the SAP region excluded the mountain area with an altitude >2000 m. We calculated the spatial distribution of the long-term trend using Theil-Sen’s trend^82^ and tested the significance of the trend using the Mann–Kendall test^83^ (Figs. 1c, 2b–g, 3 and Supplementary Figs. 1–3).

#### Anthropogenic forcing effects

The anthropogenic forcing effects on climate variability in South America were calculated as the differences between the historical simulations driven by the observed and natural-only forcing. The long-term trend of the multi-model ensemble-mean differences in the 925 hPa relative humidity, surface temperature, and surface wind speed during the 2003–2014 dry season were calculated (Fig. 2; the results for individual models are given in Supplementary Figs. 1–3). We selected the time period 2003–2014 to estimate the anthropogenic forcing effects on the SAP region because the historical simulations in DAMIP end in 2014. We also considered the late 21st century when anthropogenic forcing signals were larger by comparing the last 40 years of SSP585 (2061–2100) with the historical simulations (1975–2014) in Supplementary Figs. 7–9.

#### Deforestation

Referring to the method in Vargas Zeppetello, et al.^54^, for each year in the period of 2003–2019, we defined a pixel in the specified year as deforested if >15% of the 30-m resolution land surface grids of the 21st-Century Forest Cover Change^55^ showed deforestation activity in the particular (0.1° × 0.1°) pixel of GFAS and the other pixels as non-deforested. We used a threshold of 15%, not the 50% used previously^54^, to count as much burnt carbon in the deforested region as possible.

#### Mechanism of linkage between the AMO and fire risk in the SAP region

Based on the AMO index calculated via the Residual method, we compared the differences in the detrended variables between the negative (1969–1996, 2015–2019) and positive (1960–1968, 1997–2014) AMO years during the dry season in the time period 1960–2019. We used these results to estimate the effects of a weakening positive AMO phase on fire emissions in the SAP region. To further verify the impacts of the AMO, we applied the data processing method described here to the 925 hPa relative humidity and 850 hPa wind vectors from the 20th Century Reanalysis V3 and JRA55 datasets and the surface relative humidity from 18-model historical simulations, respectively, for the dry seasons of 1960–2015, 1960–2019, and 1850–2014 (Supplementary Figs. 5, 6).

#### Drought index

The 5-month SPEI was used to measure drought, which was calculated using the 5-month precipitation and potential evapotranspiration before the initial time (e.g., April–August for 5-month SPEI in August) from CRU4.04^84^ datasets. The smaller the value, the more severe the drought^67^.

## Supplementary information

Supplementary Information

## Data Availability

All the datasets used in this paper are publicly available. HadISST1 is available at http://hadobs.metoffice.com/hadisst/data/. EN4 is available at www.metoffice.gov.uk/hadobs/en4/. AOD from MODIS is available at https://modis.gsfc.nasa.gov/. GFAS wildfire product from the European Centre for Medium-Range Weather Forecasts is available at https://apps.ecmwf.int/datasets/data/. All the model simulations from CMIP6 are available at https://esgf-node.llnl.gov/projects/cmip6/. NCEP-R1, NCEP-R2, 20th Century Reanalysis V3, the Kaplan Extended SST version2, and AMO index from NOAA are available at https://psl.noaa.gov/data/. CRU4.04 is available at https://www.cru.uea.ac.uk/data/. JRA55 is available at https://climatedataguide.ucar.edu/climate-data/jra-55. High-Resolution Global Forest Change is available at https://developers.google.com/earth-engine/datasets/catalog.

## Code availability

The data were processed by the NCAR Command Language and Python. All the scripts are available from the corresponding authors for specific code requests.
