# Supplementary material for: Potential fire risks in South America under anthropogenic forcing hidden by the Atlantic Multidecadal Oscillation
Source: Nat Commun. 2022 May 4;13:2437. doi: 10.1038/s41467-022-30104-1 (PMC9068914; doi:10.1038/s41467-022-30104-1)
Supplement: Supplementary file 1 — Supplementary Information [file 41467_2022_30104_MOESM1_ESM.pdf]

Supplementary Material for

Potential fire risks in South America under anthropogenic forcing hidden by the Atlantic Multidecadal Oscillation

Yanfeng Wang[1,2] and Ping Huang*[1,3]

[1]Center for Monsoon System Research, Institute of Atmospheric Physics, Chinese Academy of Sciences, Beijing, China,

[2]College of Earth Sciences, University of Chinese Academy of Sciences, Beijing, China

[3]State key Laboratory of Numerical Modeling for Atmospheric Sciences and Geophysical Fluid Dynamics, Institute of Atmospheric Physics, Chinese Academy of Sciences, Beijing, China

Correspondence to Dr. Ping Huang (huangping@mail.iap.ac.cn)

Supplementary Tables

Supplementary Table 1. Annual cumulative amount of burnt carbon globally and in the SAP region and their ratio during the time period 2003–2019.

Year	Total global amount of burnt carbon (units: Tg)	Amount of burnt carbon in the SAP region (units: Tg)	Ratio (%)
2003	2440.58	336.41	14
2004	2294.52	409.97	18
2005	2242.55	398.10	18
2006	2161.88	261.04	12
2007	2251.08	440.71	20
2008	2037.19	225.19	11
2009	1908.48	144.68	8
2010	1984.17	401.18	20
2011	2105.21	182.39	9
2012	2313.20	237.33	10
2013	1912.87	140.26	7
2014	2032.27	159.74	8
2015	2329.03	207.75	9
2016	1971.46	190.71	10
2017	1753.99	211.29	12
2018	1727.24	115.00	7
2019	2100.68	231.39	11

Supplementary Table 2. Cumulative amount of burnt carbon in the SAP region and South America and their ratio during the dry season in the time period 2003–2019.

Year	Amount of burnt carbon in South America (units: Tg)	Amount of burnt carbon in the SAP region (units: Tg)	Ratio (%)
2003	256.49	236.38	92
2004	315.64	297.44	94
2005	332.28	317.38	96
2006	218.66	198.90	91
2007	384.86	371.73	97
2008	181.94	164.95	91
2009	115.84	96.09	83
2010	341.66	330.82	97
2011	152.67	140.36	92
2012	200.81	188.61	94
2013	113.95	97.96	86
2014	132.32	122.34	92
2015	170.88	156.81	92
2016	147.43	135.07	92
2017	180.43	166.52	92
2018	93.79	81.37	87
2019	194.17	179.16	92

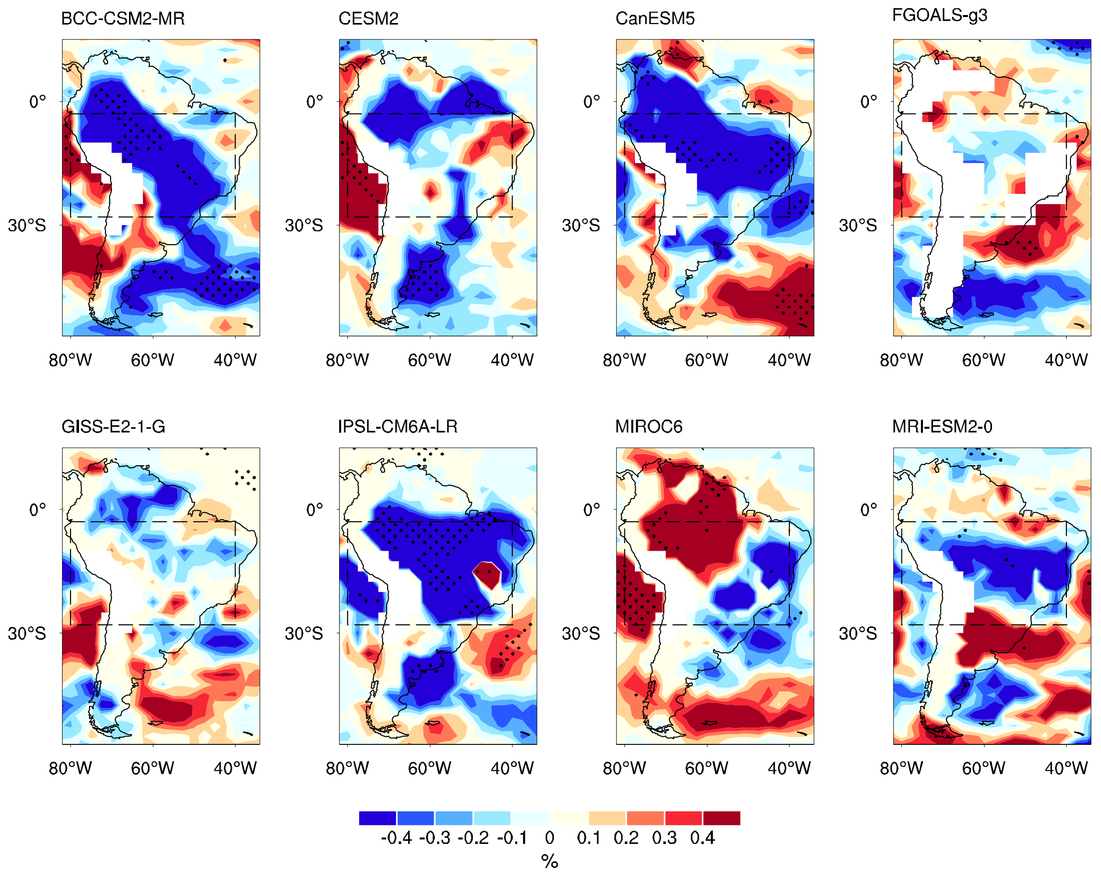

Supplementary Figure 1. Long-term trends of the 925 hPa relative humidity for the models in the DAMIP during the time period 2003–2014. Stippling indicates passing the Mann–Kendall test at the 0.1 significance level.

Supplementary Figures

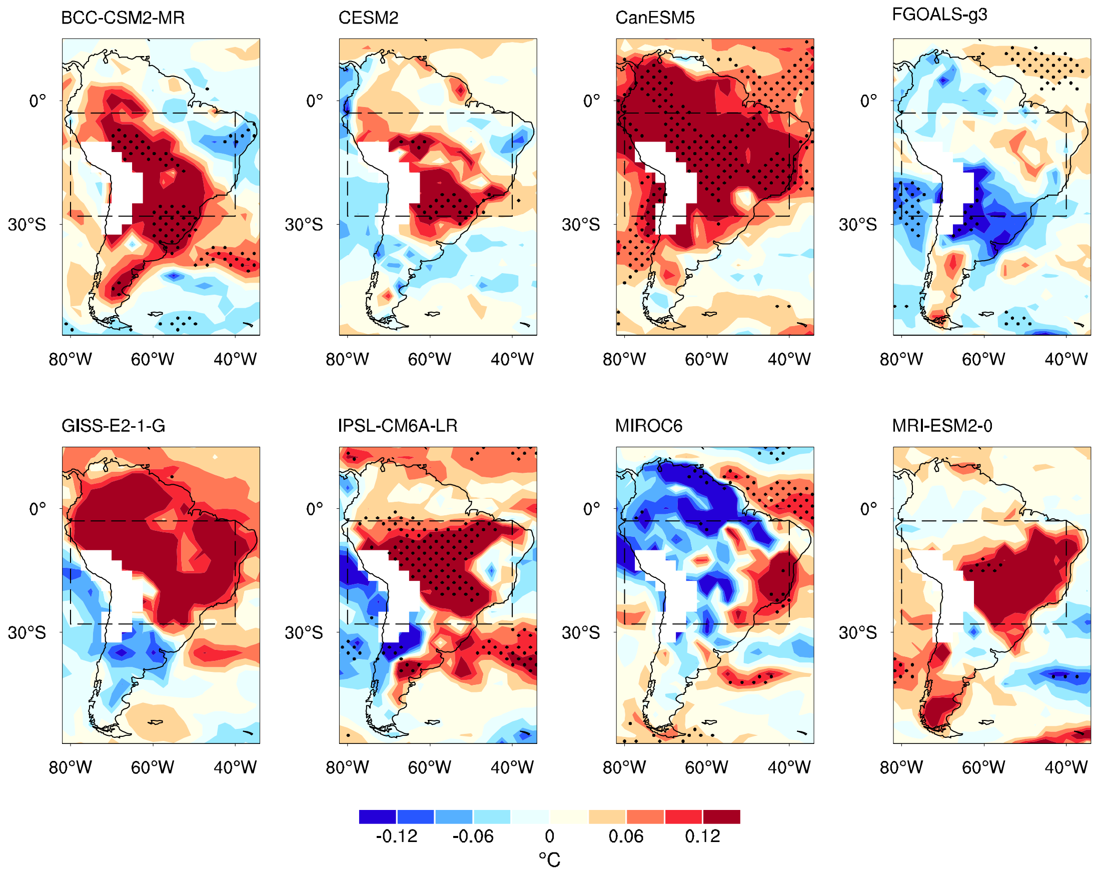

Supplementary Figure 2. Long-term trends in the surface temperature for the models in the DAMIP during the time period 2003–2014. Stippling indicates passing the Mann–Kendall test at the 0.1 significance level.

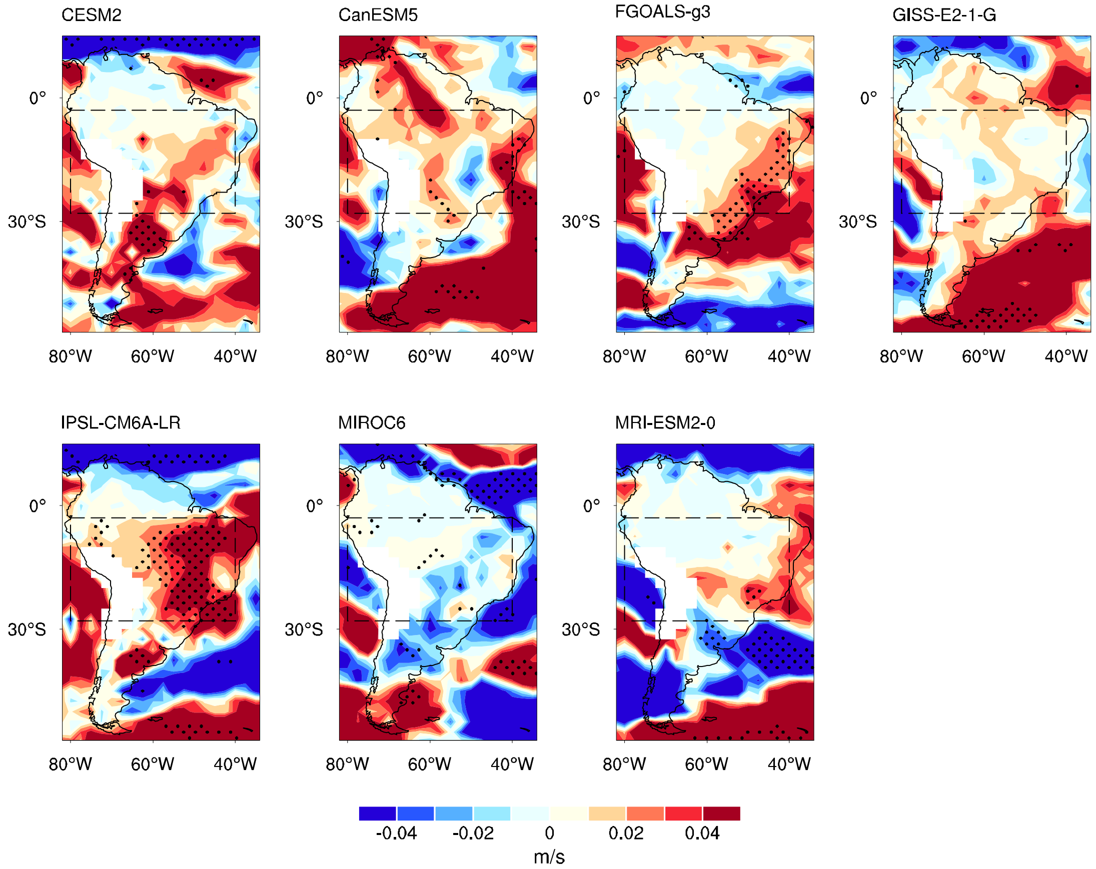

Supplementary Figure 3. Long-term trends in the surface wind speed for the models in the DAMIP during the time period 2003–2014. Stippling indicates passing the Mann–Kendall test at the 0.1 significance level.

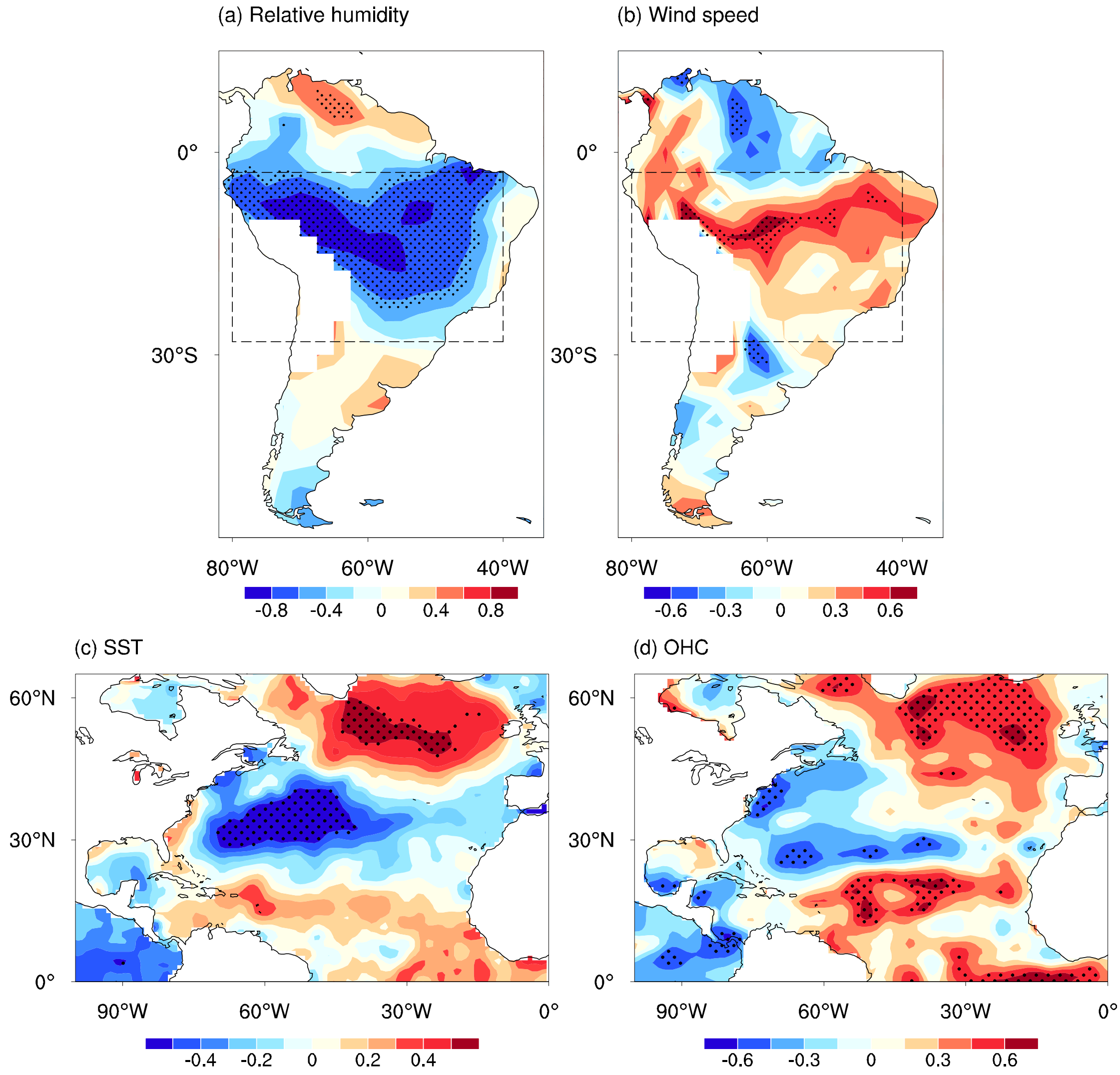

Supplementary Figure 4. Correlation coefficients of the (a) 925 hPa relative humidity, (b) surface wind speed, (c) SST and (d) OHC with the AOD in the SAP region during the dry season in the time period 2003–2019. Stippling indicates passing Student's *t*-test at the 0.05 significance level.

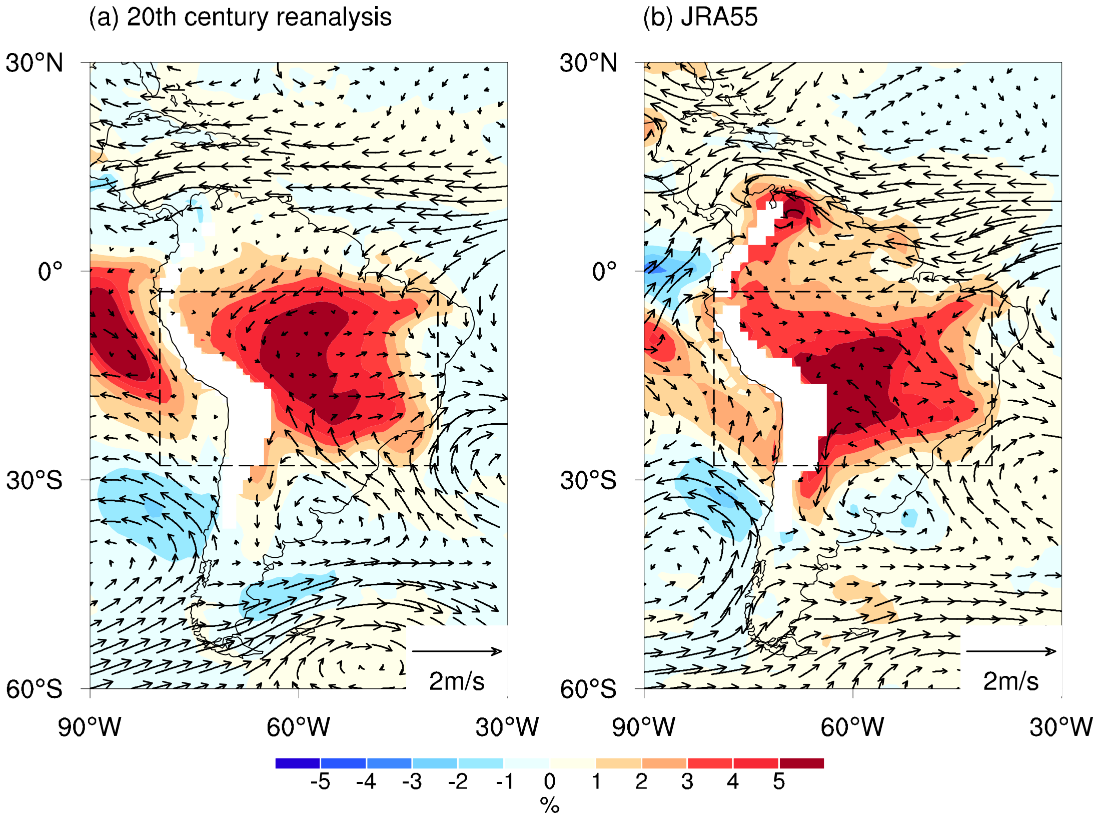

Supplementary Figure 5. Differences in the mean 925 hPa relative humidity and 850 hPa wind vectors between the negative and positive phases of the AMO in the dry season based on the 20th Century Reanalysis V3 and JRA55 datasets. The AMO index is detrended via the Residual method. The time period is 1960–2015 for the 20th Century Reanalysis V3 dataset and 1960–2019 for the JRA55 dataset.

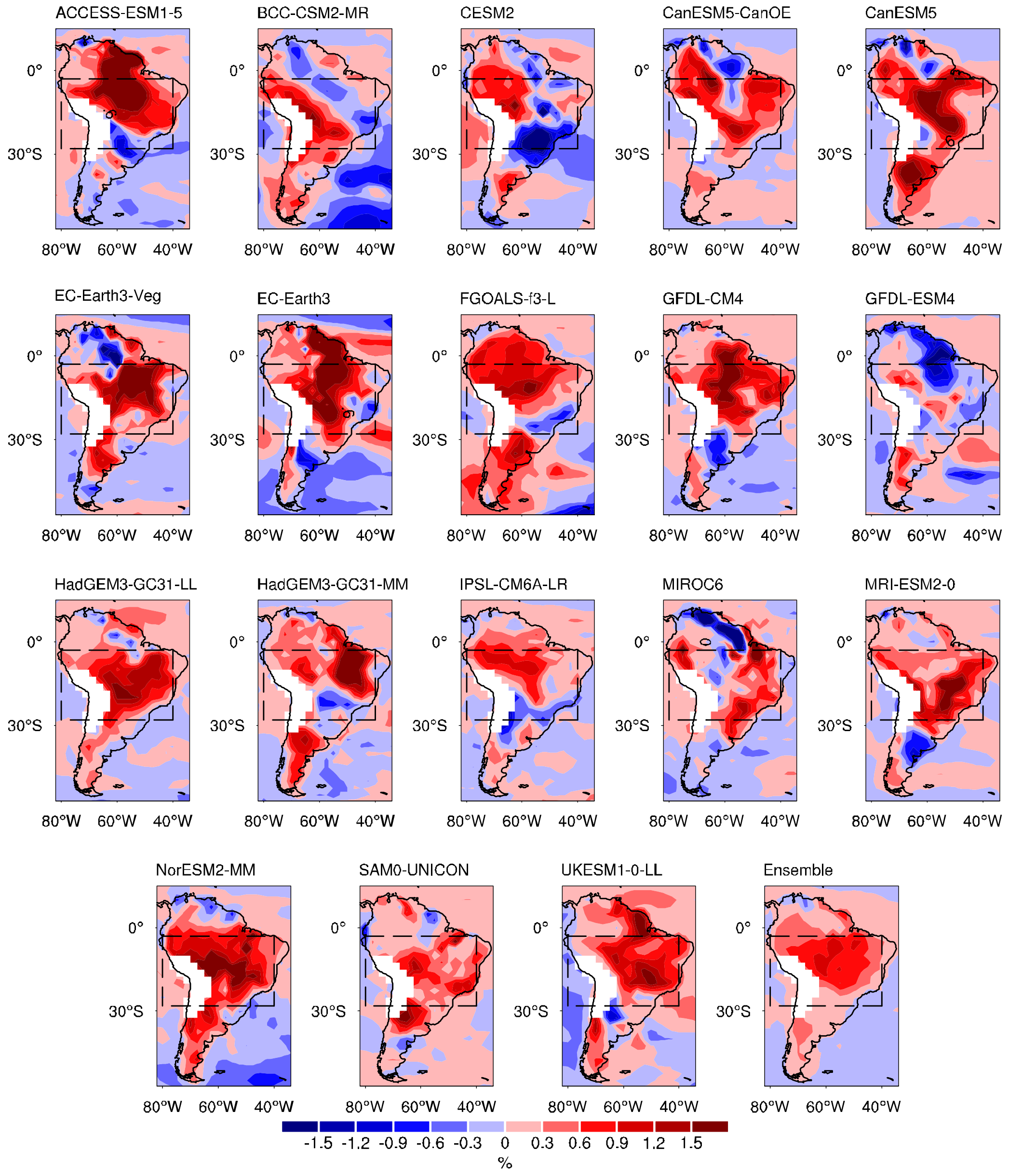

Supplementary Figure 6. Differences in the surface relative humidity between the negative and positive phases of the AMO from historical simulations of 18 CMIP6 models during the dry season in the time period 1850–2014 after applying the eight-year running mean. The AMO index is detrended via the Residual method. The surface relative humidity is removed of the linear trend in the time period 1850–2014. The SAP region is marked by the black dashed box.

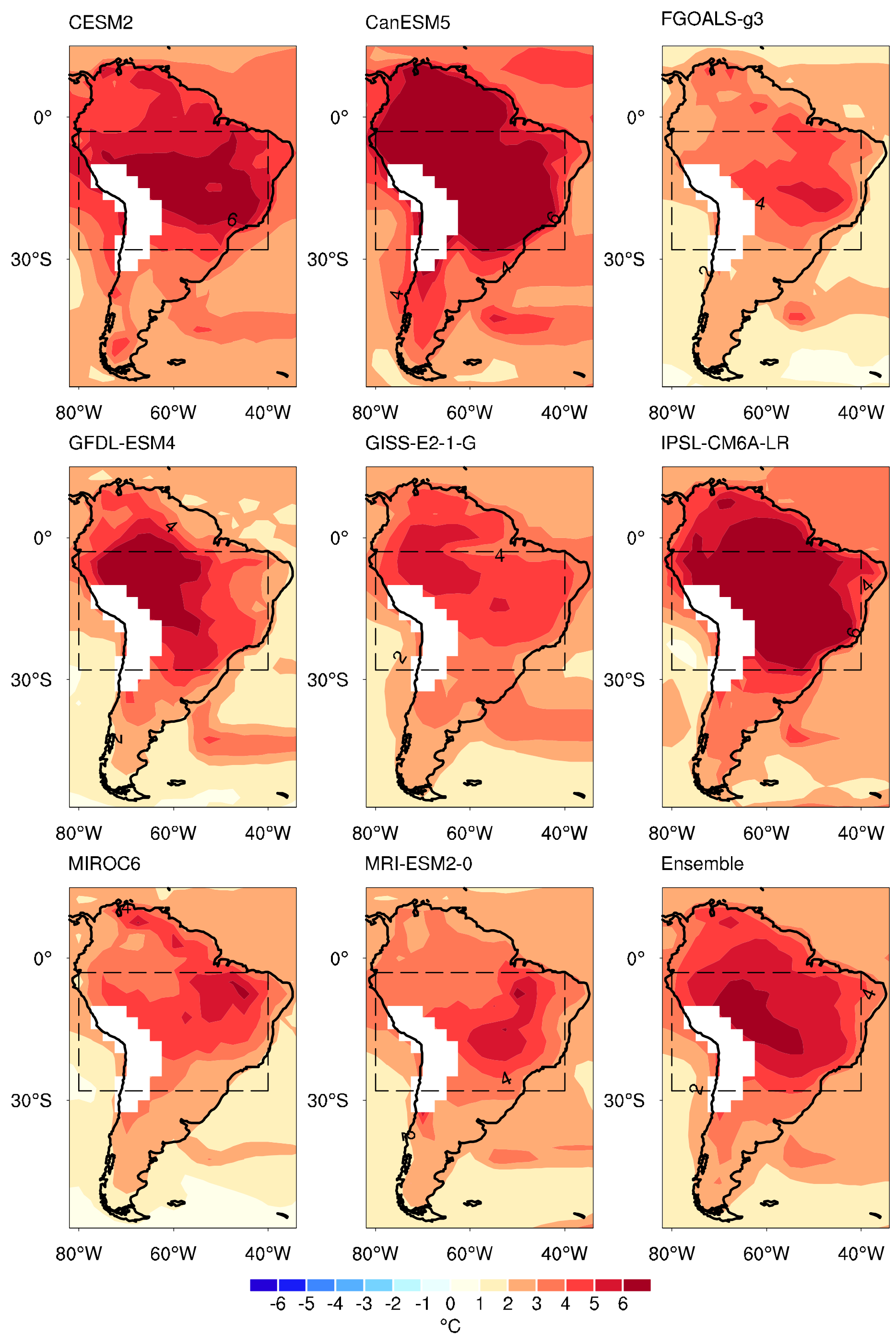

Supplementary Figure 7. Surface temperature changes in the dry season under the SSP585 scenario (2061–2100) relative to the historical simulation (1975–2014) projected by eight CMIP6 models and their ensemble mean.

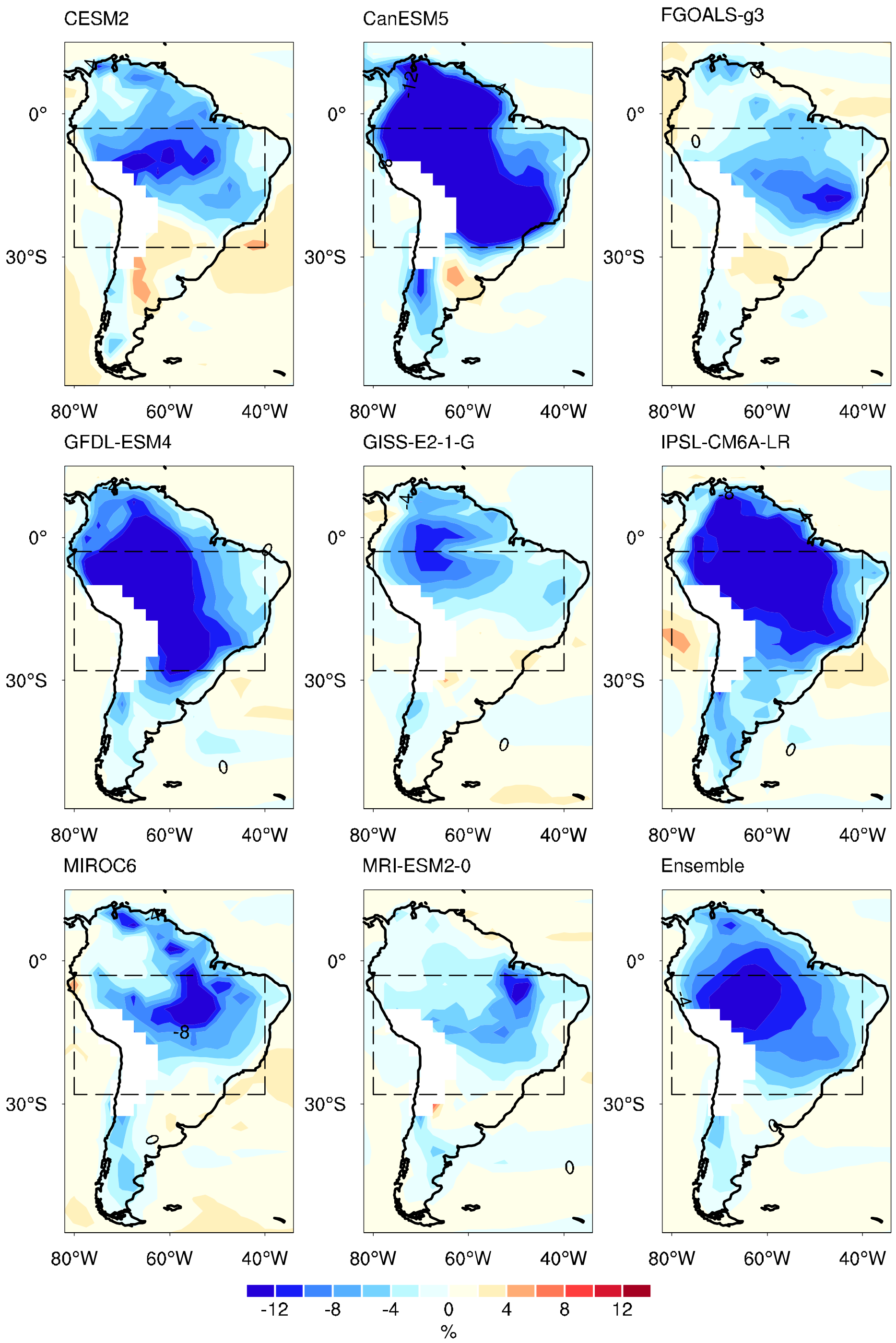

Supplementary Figure 8. Surface relative humidity changes in the dry season under the SSP585 scenario (2061–2100) relative to the historical simulation (1975–2014) projected by eight CMIP6 models and their ensemble mean.

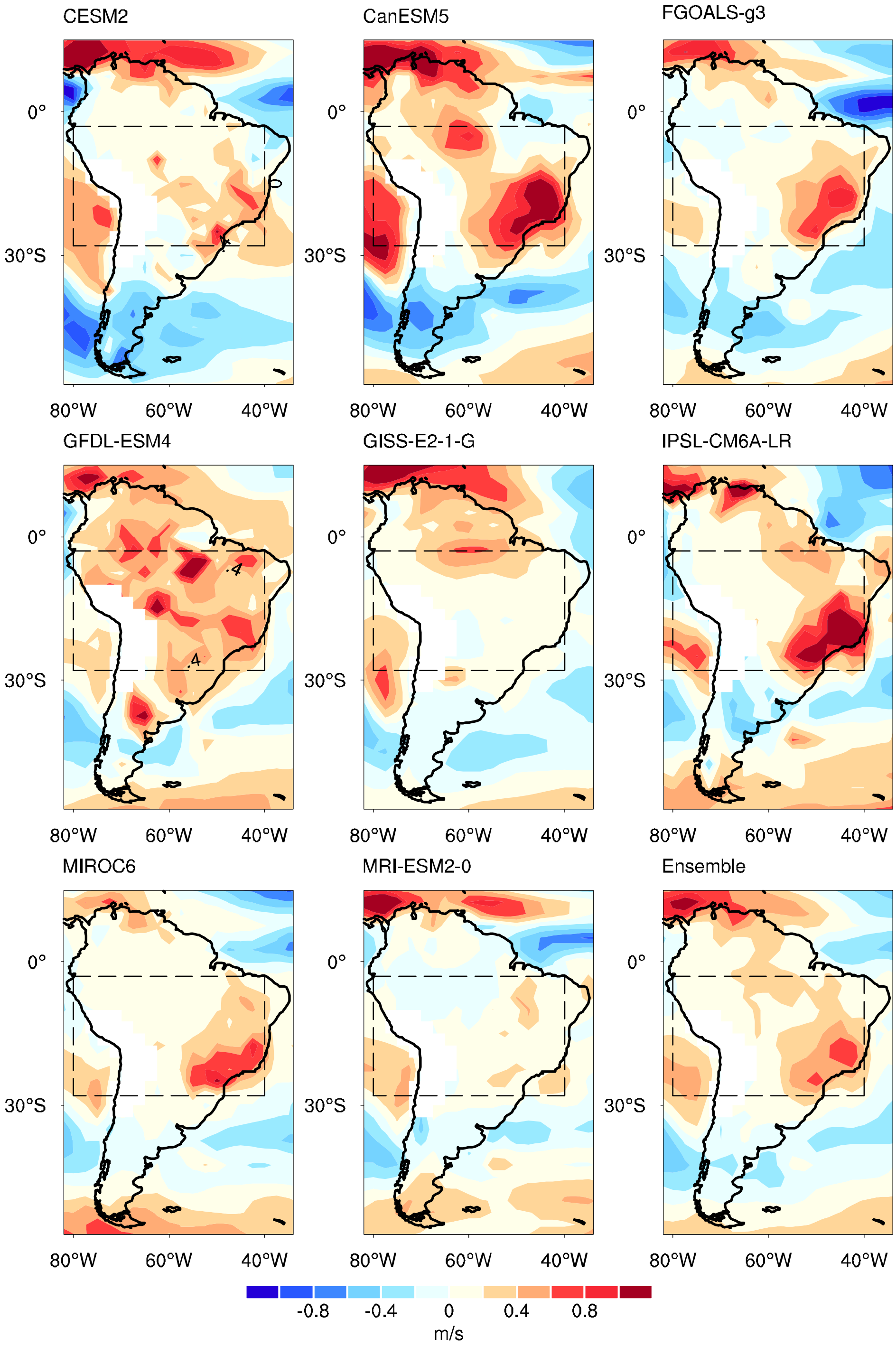

Supplementary Figure 9. Surface wind speed changes in the dry season under the SSP585 scenario (2061–2100) relative to the historical simulation (1975–2014) projected by eight CMIP6 models and their ensemble mean.

Supplementary Discussion

The calculation of AMO index

The AMO indices using the linear detrending method are different when removing the linear trend of the two different time periods (1960–2019 and 1856–2019). This indicates that the linear detrending method partly depends on the subjective selection of the detrending period. The linear detrending method can only remove part of the global warming signal in the 21st century because this signal is nonlinear [81]. To avoid these shortcomings in the linear detrending method, we removed the global mean SSTA[81] (indicated by GM) and the regression of SSTA on the yearly global mean SSTA[82] (indicated by Residual) to calculate AMO indices shown in Fig. S10. Both the AMO indices showed a decreasing tendency from the beginning of the 21st century. We also examined the influence of the dataset sources. When the linear trend in the time period 1960–2019 was removed, the AMO indices based on the HadISST1, Kaplan SST and NOAA datasets were highly consistent with each other and all showed a recent decreasing tendency.

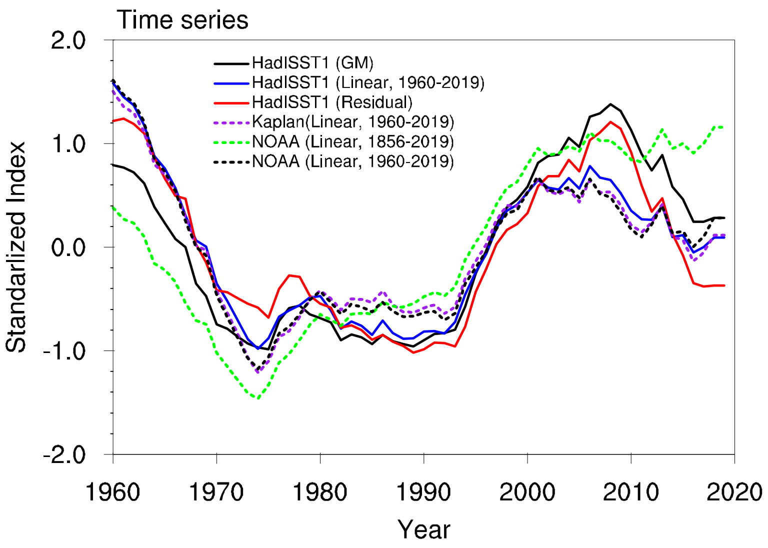

Supplementary Figure 10. Standardized annual mean AMO indices calculated by different detrending methods during the time period 1960–2019 after applying the eight-year running mean. GM indicates removal of the global mean SSTA; Linear indicates removal of the linear trend of each grid, including the linear trend for the time periods 1960–2019 and 1856–2019; Residual indicates removal of the regression of the yearly global mean SSTA time series on the SSTA.
